# Complete Genome Sequences of *Kinneretia* sp. Strain XES5, *Shinella* sp. Strain XGS7, and *Vogesella* sp. Strain XCS3, Isolated from Xenopus laevis Skin

**DOI:** 10.1128/MRA.01050-21

**Published:** 2021-12-16

**Authors:** D. T. Hudson, P. A. Chapman, R. C. Day, X. C. Morgan, C. W. Beck

**Affiliations:** a Department of Zoology, University of Otago, Dunedin, New Zealand; b Department of Biochemistry, University of Otago, Dunedin, New Zealand; c Department of Microbiology and Immunology, University of Otago, Dunedin, New Zealand; University of Arizona

## Abstract

Here, we report the genome sequences of three bacterial isolates, *Kinneretia* sp. strain XES5, *Shinella* sp. strain XGS7, and *Vogesella* sp. strain XCS3, which were cultured from skin of adult female laboratory-bred Xenopus laevis.

## ANNOUNCEMENT

Xenopus laevis (African clawed frog) is a widely used model for gene expression, vertebrate development, regeneration, and disease (1–6). We have isolated a collection of *Xenopus*-associated bacterial isolates as part of research on tadpole regeneration (6). This report presents complete genomes for *Kinneretia* sp. strain XES5, *Shinella* sp. strain XGS7, and *Vogesella* sp. strain XCS3. To date, genomes from only 2 *Kinneretia*, 17 *Shinella*, and 9 *Vogesella* species are available from the RefSeq database; many were isolated from aquatic environments (7–17), consistent with the habitat of X. laevis.

Isolates were collected from the skin of captive-bred adult female X. laevis frogs from the University of Otago *Xenopus* colony. These *Xenopus* frogs are housed in a mains-water-recirculating aquarium system and fed twice weekly with salmon pellets. Aseptically collected swab samples were plated on Oxoid nutrient agar and incubated at 30°C for 48 h, and colonies were purified by streaking.

Default parameters were used for sequencing and assembly unless otherwise noted. Sequencing quality data are summarized in Table 1. For sequencing of each isolate, single colonies were streaked on a plate. From this plate, approximately 20 individual colonies were picked and placed in lysis buffer. Isolate DNA was then extracted using a Presto Mini genomic DNA (gDNA) bacteria kit. This DNA was used for both Nanopore and Illumina sequencing. Nanopore libraries were prepared with a rapid barcoding kit (SQK-RBK004; Oxford Nanopore Technologies [ONT]) and sequenced using a GridION system (flow cell R9.4.1). Demultiplexing and base calling used Guppy (v4.3.4). Porechop (v0.2.4) (18) and Filtlong (v0.2.1) (--keep_percent 95) (19) were used to trim adapters and filter reads.

**TABLE 1:** Summary of isolate characteristics and most closely related strains by ANI

Parameter	Data for:		
	*Kinneretia* sp. strain XES5	*Shinella* sp. strain XGS7	*Vogesella* sp. strain XCS3
Genome size (bp)			
Total	5,810,938	5,248,830	3,889,382
Chromosome	4,026,671	4,025,507	3,724,844
Plasmid(s)	895,176, 733,869, 102,922, 33,339, 18,961	69,886, 18,961	164,538
G+C content (%)	68	64	60
CheckM completeness (%)	99.68	100	99.57
CheckM contamination (%)	0.39	0.58	0.11
No. of protein-coding genes	5,466	4,587	3,570
No. of RNA genes	65 (3 rRNA operons)	93 (6 rRNA operons)	136 (9 rRNA operons)
BioSample accession no.	SAMN21437801	SAMN21437802	SAMN21437800
SRA accession no.	SRR15901072, SRR15901073, SRR15901074	SRR15901069, SRR15901070, SRR15901071, SRR15901077	SRR15901075, SRR15901076, SRR15901078, SRR15901079
GenBank accession no.	GCA_020535545.1	GCA_020535565.1	GCA_020616155.1
Landcare accession no.	ICMP 24357	ICMP 24358	ICMP 24364
No. of Illumina reads	1,074,001	1,807,749	1,028,939
No. of Nanopore reads	63,881	82,808	389,151
Genome coverage (×)			
Illumina	67	98	106
Nanopore	85	107	347
Total	152	205	453
Nanopore read *N*_50_ (bp)	13,683	15,020	6,172
Most closely related genomes (ANI [%])	Kinneretia asaccharophila DSM 25082 (7) (GenBank accession no. ASM436240v1) (89.91), *Kinneretia* sp. strain DAIF2 (8) (GenBank accession no. ASM1562442v1) (85.83), *Mitsuaria* sp. strain WAJ17 (GenBank accession no. ASM1417451v1) (81.87), *Pelomonas* sp. strain KK5 (34) (GenBank accession no. ASM198409v1) (81.32), *Mitsuaria* sp. strain TWR114 (35) (GenBank accession no. ASM799734v1) (81.17)	Shinella zoogloeoides DSM 287 (GenBank accession no. ASM982685v1) (88.53), Shinella zoogloeoides PQ7 (GenBank accession no. ASM357462v1) (87.88), *Shinella* sp. strain PSBB067 (GenBank accession no. ASM1683914v1) (87.86), *Shinella* sp. strain HZN7 (15) (GenBank accession no. ASM165256v1) (87.61), Shinella granuli DSM 18401 (36) (GenBank accession no. ASM434188v1) (87.59)	Vogesella mureinivorans 389 (12) (GenBank accession no. ASM764403v2) (90.37), *Vogesella perlucida* DS-28 (9) (GenBank accession no. ASM784415v2) (90.18), *Vogesella urethralis* YM-1 (37) (GenBank accession no. ASM764404v2) (88.28), *Vogesella* sp. strain LIG4 (38) IMG taxon (GenBank accession no. 2517093049) (82.86), Vogesella alkaliphila KCTC 32041 (39) (GenBank accession no. ASM1465247v1) (82.63)

Illumina libraries were prepared with a Nextera XT kit and sequenced on a MiSeq system using both 1 × 300-bp single-end reads and 2 × 300-bp paired-end reads. Fastp (v0.20.1) (--detect_adapter_for_pe) (20) was used for adapter trimming and quality control (QC).

Reads passing QC were assembled with Trycycler (v0.5.0) (21) using Raven (v1.6.0) (22), Flye (v2.9-b1768) (23), and miniasm (v0.3-r179) (24). All assembled sequences were successfully circularized by Trycycler with the “reconcile” command. Plasmids are defined as circularized extrachromosomal sequences. Assemblies were polished with medaka (v1.4.4; ONT) and Pilon (v1.24) (25). Quality was assessed with CheckM (v1.1.3) (26); all genomes were >99% complete and had <1% contamination.

Sequences of the 16S rRNA genes were extracted using Barrnap (v0.9) (27) and compared to the NCBI 16S rRNA database (28) using BLASTn (29) to determine the five most closely related genera. Genomes from these genera were downloaded from RefSeq and compared to the isolates using FastANI (v1.32) (30) (Table 1). Genomes were uploaded to GenBank and annotated by PGAP (31, 32).

Currently, few other genomes from these genera (2 *Kinneretia* species, 17 *Shinella* species, and 9 *Vogesella* species) are available from RefSeq, and the ANI values for all three isolates are well below the 96% ANI threshold that is widely used to indicate species boundaries (33). Therefore, these genomes provide a valuable addition to our knowledge of these three genera.

### Data availability.

All sequencing data have been deposited in DDBJ/ENA/GenBank and the SRA under BioProject PRJNA763310. Isolates have been deposited at Manaaki Whenua-Landcare Research (New Zealand). Accession numbers are shown in Table 1.
